# Septooptic Dysplasia with an Associated Arachnoid Cyst

**DOI:** 10.1155/2016/5493769

**Published:** 2016-11-06

**Authors:** Skyler V. McLaurin-Jiang, Julie K. Wood, David F. Crudo

**Affiliations:** ^1^Department of Pediatrics, Wake Forest School of Medicine, Winston-Salem, NC, USA; ^2^Section on Hospital Medicine, Department of Pediatrics, Wake Forest School of Medicine, Winston-Salem, NC, USA; ^3^Section on Endocrinology, Department of Pediatrics, Wake Forest School of Medicine, Winston-Salem, NC, USA

## Abstract

A 4-week-old male infant presented with hypothermia, hypoglycemia, and hyperbilirubinemia. His medical history was remarkable for hydrocephalus secondary to an arachnoid cyst, intermittent hypoglycemia, hypothermia, and poor feeding requiring nasogastric tube for nutrition. Physical exam revealed retrognathia, mild hypotonia, micropenis, and clinodactyly. Ophthalmologic exam demonstrated bilateral optic nerve hypoplasia (ONH). Laboratory data confirmed inadequate cortisol and growth hormone response to hypoglycemia, a low thyroxine level, and direct hyperbilirubinemia. Magnetic resonance imaging of the brain confirmed the known history of arachnoid cyst with hydrocephalus but also revealed anterior pituitary hypoplasia, absence of the posterior pituitary bright spot, a thin pituitary stalk, and bilateral optic nerve hypoplasia. A diagnosis of septooptic dysplasia (SOD) was made. Hormone replacement with hydrocortisone and levothyroxine was started with improvement in the infant's glycemic control, thermoregulation, feeding, and cholestasis. This case reinforces the importance of careful physical examination and laboratory review in a patient with known history of arachnoid cyst which has been previously described as an associated feature of optic nerve hypoplasia and hypopituitarism.

## 1. Introduction

Septooptic dysplasia (SOD), previously known as de Morsier syndrome, is a rare developmental disorder with an incidence of 1 in 10,000 [1]. The classic triad is optic nerve hypoplasia (ONH), hypopituitarism, and absence of the septum pellucidum. Traditionally, a diagnosis of SOD can be made if the patient manifests two of the three features. Approximately 30% of the time, patients have all three defects [2].

SOD can be associated with other midline brain abnormalities beyond those described in the classic triad. In particular, intracranial primary arachnoid cysts have been described in association with SOD.

We report a case of SOD, with bilateral ONH, hypopituitarism, and an associated arachnoid cyst. Although the patient had recurrent hypoglycemia, hypothermia, and poor feeding for which neuroimaging was performed shortly after birth, the diagnosis of SOD was not made until 4 weeks of age when the physical exam and subsequent clinical course prompted an endocrinologic evaluation and review of the initial magnetic resonance imaging (MRI).

This case report emphasizes the importance of careful review of neuroimaging, appropriate laboratory evaluations, and careful physical examination to facilitate a timely diagnosis of SOD.

## 2. Case Presentation

A 4-week-old Caucasian male with hydrocephalus secondary to an arachnoid cyst was admitted to our institution for concerns of neonatal sepsis.

Patient was a 2.96 kg product of a term pregnancy delivered vaginally with forceps assistance, to a 19-year-old G1P1, serology negative, Group B Streptococcus positive (treated with penicillin), blood type O Rh (+) mother. He was apneic at birth and required positive pressure ventilation. Apgar scores were 4 at 1 minute and 9 at 5 minutes. He was admitted to the Neonatal Intensive Care Unit for hypothermia, hypoglycemia, hypotension, apneic events, and jaundice.

No physical abnormalities were noted upon admission. He received seven days of antibiotics for presumed neonatal sepsis and five days of phototherapy for hyperbilirubinemia secondary to ABO incompatibility (infant blood type was A Rh (+) and was direct antiglobulin test positive).

A cranial ultrasound was performed at 1 week of age due to recurrent temperature instability, hypoglycemia, and poor feeding, revealing an arachnoid cyst in the right hemisphere. MRI of brain was obtained which showed hydrocephalus and a midline shift secondary to the obstructing arachnoid cyst. The patient was then transferred to our hospital. Neurosurgery was consulted, and he underwent fenestration of the arachnoid cyst. He improved clinically but had continued feeding difficulties for which nasogastric feeds were continued at time of discharge on day of life 29.

At 4 weeks of age (two days after prior discharge), he presented to our Emergency Department with hypothermia (35.1°C, rectally) and hypoglycemia (2.5 mmol/L). Cerebrospinal fluid, urine, and blood cultures were obtained and he received intravenous ampicillin, cefotaxime, and acyclovir for 48 hours. All cultures were ultimately negative.

Physical exam was notable for retrognathia, micropenis of 1.8 cm stretched length, and clinodactyly. We pursued further endocrinologic workup due to concern for hypopituitarism considering his hypoglycemia and associated midline defects. A “critical blood sample” was obtained at a time of hypoglycemia and a glucagon stimulation test was performed. The results are presented in Table 1.

Serum levels at a time of hypoglycemia were remarkable for decreased cortisol and growth hormone responses. The insulin level was appropriately suppressed. Free fatty acids and *β*-hydroxybutyrate levels were low. Lactic acid, acylcarnitine profile, plasma amino acids, and urine organic acids were normal. A glucagon stimulation test showed a normal glucose response. Thyroid function tests were notable for borderline free T4 and normal thyroid stimulating hormone (TSH) levels.

His total bilirubin was 70.1 *μ*mol/L (1.7–20.5) with a direct fraction of 25.7 *μ*mol/L (1.7–3.4). Transaminase levels were normal and ammonia was mildly elevated. An abdominal ultrasound did not demonstrate any hepatobiliary abnormalities. Deoxyribonucleic acid microarray was unremarkable.

Ophthalmologic examination revealed bilateral hypoplastic optic nerves.

**Figure 1:**
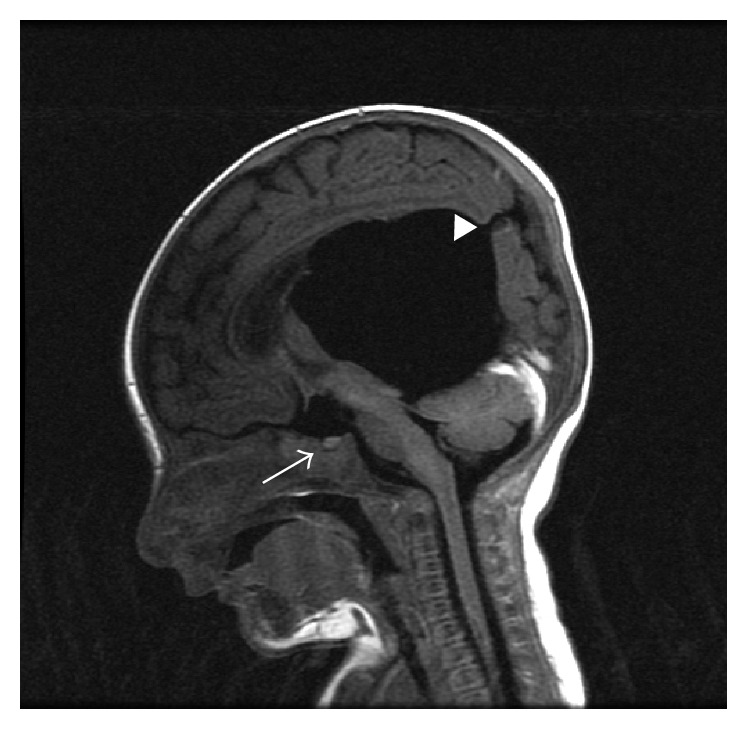
The white arrow indicates the hypoplastic anterior pituitary. Note that the posterior pituitary bright spot is not seen. The solid white triangle shows the arachnoid cyst status after fenestration.

**Figure 2:**
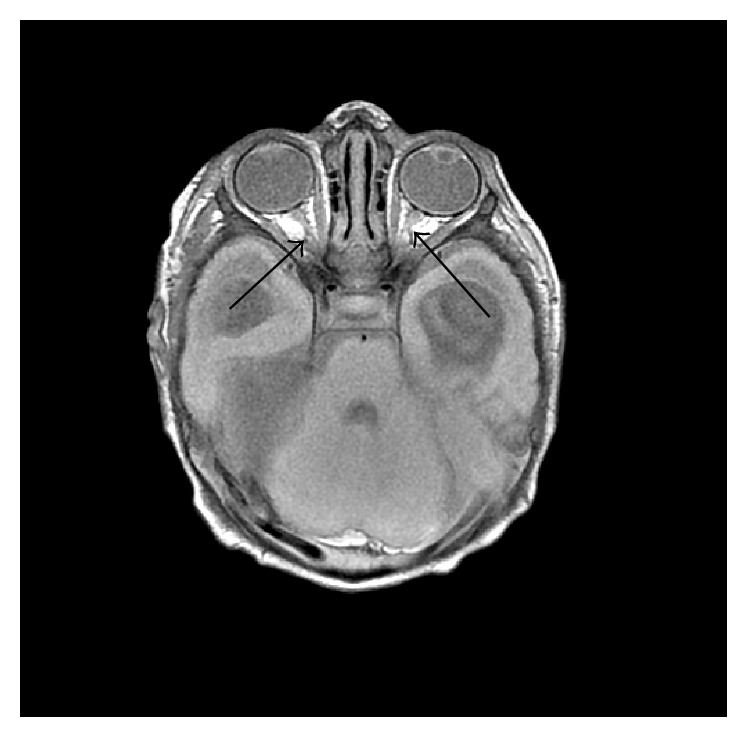
The black arrows indicate the hypoplastic optic nerves.

Neuroradiology review of his previous brain MRI from outside institution confirmed hypoplastic optic nerves, small anterior pituitary with thin infundibulum, lack of a posterior pituitary bright spot, intact corpus callosum and septum pellucidum, and the previously described arachnoid cyst and hydrocephalus (Figures 1 and 2).

The inadequate hormonal response to hypoglycemia, secondary hypothyroidism, and an abnormal pituitary gland on imaging was consistent with the diagnosis of multiple pituitary hormone deficiencies (panhypopituitarism). Hydrocortisone and levothyroxine replacement therapy were initiated that achieved euglycemia and temperature stability.

## 3. Discussion

One of the main presenting features in this neonate was hypoglycemia, for which there is a long differential diagnosis. In this case, however, the infant's concomitant micropenis, hypothermia, and poor energy and tone point to underlying hypothalamic-pituitary dysfunction. The infant was closely monitored in a fasting state, and a diagnostic critical blood sample was obtained when he was hypoglycemic, followed by a glucagon stimulation test. Results from the critical sample confirmed deficient cortisol and growth hormone responses. In addition, the infant was found to have a nonketotic hypoglycemia which is typically seen in hyperinsulinism or disorders of free fatty acid oxidation. However, nonketotic hypoglycemia can also be seen in hypopituitarism and is included in the list of “hyperinsulinism mimickers” [3, 4]. The infant had an appropriately suppressed insulin level during his hypoglycemic event, and free fatty acid levels were normal. The glucagon stimulation test also showed a normal response.

Further evaluation of the pituitary axis revealed borderline thyroid function. The gonadal axis was not tested at this time due to the patient's age. Gonadotropin and testosterone deficiency was presumed in light of his abnormally short penile length. His urine output and serum electrolytes were closely monitored during the hospital stay, and he exhibited no signs of vasopressin deficiency. The results of his testing and physical findings are consistent with the diagnosis of panhypopituitarism.

Cholestasis, defined by a direct bilirubin level >20% of the total serum bilirubin level and seen in this case, has been associated with hypopituitarism [5, 6]. Cholestasis typically represents an underlying pathologic condition, with a wide differential including infections, anatomic abnormalities of the biliary system, toxic exposures, oncologic processes, and endocrine and metabolic disorders [7]. An abdominal ultrasound did not identify any findings of an extrahepatic obstructive lesion (choledochal cyst, mass, gallstone, and sludge).

The mechanism responsible for the development of cholestasis in hypopituitarism is not clear. Since growth hormone and cortisol play a role in bile acid synthesis and biliary flow it has been postulated that deficiencies in these hormones may be the underlying cause of the cholestasis [8]. The cholestasis typically resolves in several months after hormone replacement has been initiated. In our case, the patient's direct bilirubin resolved with hormonal replacement.

There are a multitude of associated neuroradiological anomalies detectable by MRI in cases of SOD. In this case, a large arachnoid cyst was noted on initial read of the MRI at 1 week of age. In 2004, Lyons et al. [9] reported that large arachnoid cysts are associated with up to 12.5% of cases of ONH. Arachnoid cysts commonly complicate midline malformations of the brain including agenesis of the corpus callosum and holoprosencephaly [10].

Abnormalities in the optic and olfactory tracts, infundibulum, and anterior and posterior pituitary can be subtle radiological findings and were not recognized until review of the MRI three weeks later. This patient's MRI did reveal an intact septum pellucidum, a thin infundibulum with a small anterior pituitary and absence of posterior pituitary bright spot, and thread-like optic nerves. Posterior pituitary ectopia is highly predictive of pituitary hormone deficiencies [11].

The underlying pathogenic process of SOD remains unclear but is thought to be related to the holoprosencephaly spectrum [9, 12–14]. The vast majority of cases are sporadic; however rare familial cases have suggested a connection to* HESX1* which is a homeobox gene involved in the development of the prosencephalon and is a marker of pituitary development [15]. The etiology is likely multifactorial involving interplay between environmental factors and genetic development.

Neurodevelopmental outcomes in patients with SOD are quite variable. In general, patients with SOD with unilateral ONH phenotype have better developmental outcomes than those with bilateral ONH [12]. Prompt recognition of underlying endocrinologic disturbances and appropriate treatment is associated with improved outcomes.

## 4. Conclusion

Hypoglycemia, micropenis, cholestasis, and hypothermia, especially when concurrent, are clinical signs which should heighten one's suspicion for hypopituitarism. Septooptic dysplasia is a rare, but important, cause of hypopituitarism and can be associated with other malformations of the prosencephalon. Large arachnoid cysts are typically rare and ought to prompt detailed review of neuroimaging for any associated brain malformations. Early diagnosis of septooptic dysplasia is crucial in improving neurodevelopmental outcomes by treatment of associated hormonal abnormalities.

## Figures and Tables

**Table 1:** Summary of pertinent lab values.

	Value	Units	Reference ranges
Free T4	9.0	pmol/L	7.7–22.5
TSH	3.4	*μ*IU/mL	0.40–5.50
Ammonia	**75**	*μ*mol/L	20–55
Total bilirubin	**70.1**	*μ*mol/L	1.7–20.5
Direct bilirubin	**25.7**	*μ*mol/L	1.7–3.4

Critical sample			
Glucose	**2.1**	mmol/L	3.9–6.1
Cortisol	**11.0**	nmol/L	>500^*∗*^
Growth hormone	**3.2**	*μ*g/L	>6.0^*∗*^
Insulin	0.7	pmol/L	<13.9^*∗*^
Lactate	0.8	mmol/L	0.5–2.2
Free fatty acids	**0.27**	mmol/L	>0.50^*∗*^
*β*-Hydroxybutyrate	**0.12**	mmol/L	>1.0^*∗*^
Acylcarnitine profile	Normal		
Serum amino acids	Normal		
Urine organic acids	Normal		

Glucagon stimulation test			
+10 min Glucose	4.2	mmol/L	
+20 min Glucose	5.1	mmol/L	
+30 min Glucose	5.0	mmol/L	

^*∗*^Levels expected during hypoglycemia.

Abnormal values are in **bold**.
